# Gastric Bypass Surgery Is Followed by Lowered Blood Pressure and Increased Diuresis - Long Term Results from the Swedish Obese Subjects (SOS) Study

**DOI:** 10.1371/journal.pone.0049696

**Published:** 2012-11-29

**Authors:** Peter Hallersund, Lars Sjöström, Torsten Olbers, Hans Lönroth, Peter Jacobson, Ville Wallenius, Ingmar Näslund, Lena M. Carlsson, Lars Fändriks

**Affiliations:** 1 Department of Gastrosurgical Research and Education, Institute of Clinical Sciences, Sahlgrenska Academy, University of Gothenburg, Gothenburg, Sweden; 2 Department of Molecular and Clinical Medicine and Center for Cardiovascular and Metabolic Research, Sahlgrenska Academy, University of Gothenburg, Gothenburg, Sweden; Bambino Hospital, Italy

## Abstract

**Objective:**

To compare two bariatric surgical principles with regard to effects on blood pressure and salt intake.

**Background:**

In most patients bariatric surgery induces a sustained weight loss and a reduced cardiovascular risk profile but the long-term effect on blood pressure is uncertain.

**Methods:**

Cohort study with data from the prospective, controlled Swedish Obese Subjects (SOS) study involving 480 primary health care centres and 25 surgical departments in Sweden. Obese patients treated with non-surgical methods (Controls, n = 1636 and n = 1132 at 2 y and 10 y follow up, respectively) were compared to patients treated with gastric bypass (GBP, n = 245 and n = 277, respectively) or purely restrictive procedures (vertical banded gastroplasty or gastric banding; VBG/B, n = 1534 and n = 1064, respectively).

**Results:**

At long-term follow-up (median 10 y) GBP was associated with lowered systolic (mean: −5.1 mm Hg) and diastolic pressure (−5.6 mmHg) differing significantly from both VBG/B (−1.5 and −2.1 mmHg, respectively; p<0.001) and Controls (+1.2 and −3.8 mmHg, respectively; p<0.01). Diurnal urinary output was +100 ml (*P*<0.05) and +170 ml (*P*<0.001) higher in GBP subjects than in weight-loss matched VBG/B subjects at the 2 y and 10 y follow-ups, respectively. Urinary output was linearly associated with blood pressure only after GBP and these patients consumed approximately 1 g salt per day more at the follow-ups than did VBG/B (*P*<0.01).

**Conclusions:**

The purely restrictive techniques VBG/B exerted a transient blood pressure lowering effect, whereas gastric bypass was associated with a sustained blood pressure reduction and an increased diuresis. The daily salt consumption was higher after gastric bypass than after restrictive bariatric surgery.

## Introduction

The prevalence of obesity is dramatically increasing and associated morbidities such as myocardial infarction, stroke, cancer and diabetes are serious additional demands on health care providers [1], [2]. Bariatric surgery is the only treatment that has proved to maintain a reduced body weight over time [3]. Even more important, this type of gastrointestinal surgical interference improves most of the obesity-associated cardiovascular risk factors [4]–[6]. Interestingly, several of these improvements are not dependent on the body weight reduction indicating that the gastrointestinal tract directly influences the metabolic control. Hypertension is a risk factor in the metabolic syndrome that is strongly associated with heart disease and stroke [7]. Controlled studies with short (2 y to 4 y) follow-up periods indicate that bariatric surgery exerts a blood pressure lowering effect [4], [8], [9]. However, controlled studies on the efficacy over longer periods are lacking or inconclusive [6]. The large scale, prospective, controlled Swedish Obese Subjects (SOS) study demonstrated that recovery from hypertension had improved 10y after bariatric surgery, whereas incidence had not.|4] Two factors made us reconsider the long-term results regarding blood pressure in the SOS study: 1./the study is still ongoing, and the number of 10 y follow-ups have increased since the previous report; and 2./the treatment arm in the SOS study consists of two principles for gastrointestinal interference – gastric bypass (GBP) and restrictive gastric band procedures – that could theoretically have different effects on blood pressure.

Restrictive bariatric surgery refers to a narrowing of the stomach lumen by certain banding procedures and is represented in the present study by vertical banded gastroplasty or gastric banding (VBG/B). These procedures provide a resistance (‘restriction’) to food passage into the distal part of the stomach and excessive meal results in food retention in the gastric compartment oral to the band. Distension-sensitive afferent nerves in the wall of the proximal stomach, and presumably also in the esophagus, will then mediate sensations of discomfort and even pain that consequently motivates the individual to terminate food ingestion. However, also more subtle mechanisms may be involved. Early satiation and inter-meal satiety have recently been shown to be induced with well calibrated adjustable gastric bands giving only a briefly delayed bolus transit into the infraband stomach, thus without food retention. The satiety signalling in this case is ascribed to esophageal reinforced propulsive motility and compression of gastric vagal afferents at the level of the gastric band [10]. The other principle, GBP, has more complex and partly unknown mechanisms of action. Gastric flow resistance is probably less important and instead the unloading of the gastroduodenum of nutrients and/or the direct loading of the jejunum influence appetite and improve metabolic control [11]. One aim of the present study was to investigate the effect on blood pressure of these two surgical principles. Furthermore, dietary salt (NaCl) is of great importance for body fluid homeostasis and blood pressure regulation [12]–[14]. Consequently, because both surgical and non-surgical obesity treatment regimens markedly influence food intake, it can be expected that salt ingestion is altered, which could give secondary effects on blood pressure. A second aim was thus to investigate whether, after either of the two surgical principles, changes in salt intake is important for blood pressure.

## Methods

### Study outline and ethics statement

The present investigation is based on the SOS study, which compares obese patients undergoing bariatric surgery (n = 2010) with contemporaneously matched, non-operated obese control patients (n = 2037). The inclusion of study patients began 1987 and was continued until 2001. The design and selection of the study participants have been described in detail elsewhere [4], [15]. The SOS study is conducted according to the principles for experiments with human beings as defined in the Declaration of Helsinki and the study protocol was approved by the Research Ethics Committee of University of Gothenburg (decision no S 604-01). This principal decision was also exposed to, and approved by seven other Swedish regional ethics review boards, each harbouring one or several of the involved study sites. All participants had given informed consent. Due to national recommendations when the SOS-study was launched in 1987 only verbal informed consent was initially used but later, when Sweden had adapted to the international harmonisation of ethical guidelines, written informed consents were instead used. In order to focus on effects of the two principle bariatric surgical techniques, the intention-to-treat analysis was abandoned. The present investigation was instead designed as a cohort analysis with one short-term (median 2 y, Table 1) and one long-term (median 10 y) follow-up period (Table 2).

**Table 1:** Baseline characteristics of study subjects who completed follow-up after 2 y.

	GBP	VBG/B	Control
	(P = GBP *vs* VBG/B)	(*P* = VBG/B *vs* Control)	(*P* = Control *vs* GBP)
No. of patients	245	1534	1636
Median follow-up years (mean)	2.0 (2.1)	2.0 (2.1)	2.0 (2.0)
(min to max)	(1.9 to 3.6)	(1.0 to 3.4)	(1.7 to 3.2)
Age (years)	47.1 (6.0)	47.3 (6.0)***	48.9 (6.2)***
Female sex (%)	73.05.00	70.5	70.4
Height (m)	1.68 (0.09)	1.69 (0.09)	1.69 (0.09)
Body weight (kg)	125 (19)***	120 (16)***	115 (16)***
BMI (kg/m2)	43.8 (5.0)***	42.1 (4.2)***	40.0 (4.6)***
User of anti-hypertensives (%)	39.02.00	38.7	39.9
User of diuretics (%)	22.03	19.6	18.2
**Blood pressure**			
Systolic pressure (mm Hg)	148 (21)∧	145 (18)***	138 (18)***
Diastolic pressure (mm Hg)	89 (13)	90 (11)***	85 (11)***
**Urinary (24 h)**			
Volume (L)	1.86 (0.64)	1.80 (0.65)***	1.90 (0.70)
Volume:Body weight (mL/kg)	15.2 (5.4)	15.2 (5.6)***	16·9 (6·8)***
Sodium (mmol)	229 (96)	222 (96)**	211 (90)**
Sodium:Body weight (mmol/kg)	1.86 (0.74)	1.85 (0.76)	1.84 (0.75)
Estimated daily salt intake¶ (g)	13.4 (5.6)	13.0 (5.6)**	12.3 (5.3)**
Potassium (mmol)	81 (31)	84 (31)	85 (31)∧
Sodium:Potass.	3.0 (1.3)**	2.8 (1.1)***	2.6 (1.1)***
Creatinine (mmol)	14.3 (4.6)∧	14.9 (4.5)**	14.4 (4.5)
**Serum**			
Sodium (mM)	139.6 (2.4)***	138.7 (2.6)***	139.1 (2.7)**
Potassium (mM)	4.23 (0.28)∧	4.19 (0.31)***	4.15 (0.28)***
Creatinine (µM)	86.6 (11.4)	86.5 (11.0)	86.9 (11.9)

Values are mean (±SD) unless otherwise stated. BMI, body mass index. GBP, gastric bypass surgery. VBG/B, pure restrictive bariatric surgery.

*** *P*<0.001,

** *P*<0.01,

* *P*<0.05 and ∧*P*<0.10 using Students *t* test or Chi-square test.

¶ Daily salt intake was calculated by multiplying urinary sodium values by 0.0585 (molecular weight of NaCl: 58.5).

**Table 2:** Baseline characteristics of study subjects who completed follow-up after 10 y.

	GBP	VBG/B	Control
	(P = GBP *vs* VBG/B)	(*P* = VBG/B *vs* Control)	(*P* = Control *vs* GBP)
No. of patients	277	1064	1132
Median follow-up years (mean)	10.0 (9.1)	10.0 (10.1)	10.0 (10.1)
(min to max)	(5.0 to 12.3)	(5.0 to 12.5)	(9.9 to 12.6)
Age (years)	47.1 (6.0)	47.5 (5.9)***	48.7 (6.1)***
Female sex (%)	72.9	70.1	69.8
Height (m)	1.69 (0.09)	1.69 (0.09)	1.69 (0.09)
Body weight (kg)	122 (18)**	119 (16)***	114 (16)***
BMI (kg/m2)	42.8 (4.7)**	41.9 (4.1)***	39.8 (4.6)***
User of anti-hypertensives (%)	35.3	36.4	36.7
User of diuretics (%)	16.9	17.7	15.4
**Blood pressure**			
Systolic pressure (mm Hg)	146 (20)	145 (18)***	138 (17)***
Diastolic pressure (mm Hg)	90 (12)	90 (11)***	85 (11)***
**Urinary (24 h)**			
Volume (L)	1.87 (0.74)	1.80 (0.65)**	1.88 (0.69)
Volume:Body weight (mL/kg)	15.5 (6.0)	15.3 (5.6)***	16.9 (6.6)***
Sodium (mmol)	230 (102)	224 (98)***	209 (88)**
Sodium:Body weight (mmol/kg)	1.89 (0.78)	1.87 (0.77)	1.85 (0.74)
Estimated daily salt intake¶ (g)	13.5 (6.0)	13.1 (5.7)***	12.2 (5.2)**
Potassium (mmol)	84 (31)	85 (31)	86 (31)
Sodium:Potass.	2.9 (1.3)^	2.7 (1.1)***	2.6 (1.0)***
Creatinine (mmol)	14.7 (4.6)	14.9 (4.5)*	14.6 (4.5)
**Serum**			
Sodium (mM)	139.3 (2.6)***	138.6 (2.6)***	139.1 (2.7)
Potassium (mM)	4.24 (0.29)*	4.19 (0.30)***	4.14 (0.28)***
Creatinine (µM)	86.2 (11.2)	85.9 (10.6)	86.6 (11.5)

Values are mean (±SD) unless otherwise stated. BMI, body mass index. GBP, gastric bypass surgery. VBG/B, pure restrictive bariatric surgery.

*** *P*<0.001,

** *P*<0.01,

* *P*<0.05 and ^*P*<0.10 using Students *t* test or Chi-square test.

¶ Daily salt intake was calculated by multiplying urinary sodium values by 0.0585 (molecular weight of NaCl: 58.5).

### Study groups

The SOS study subjects were sorted into the following three study cohorts (for details see Supporting information Figure S1): Gastric bypass (GBP) patients, including the patients originally operated with GBP, but also patients converted from VBG/B to GBP, as well as subjects that were originally allocated to the control arm of the SOS study but had been treated with GBP during the study period, if the GBP operation had been performed at least 5 y prior to the 10 y follow-up visit; Vertical banded gastroplasty or gastric banding (VBG/B) patients, excluding those who had been converted to GBP but including control subjects in the SOS study that had been treated with VBG/B if performed at least 5 y prior to the 10 y follow-up; Non-operated control patients, including all subjects that were originally allocated to the control arm of the SOS study and had remained on conventional treatment. Subjects with bariatric constructions other than GBP and VBG/B were excluded from the analysis. With regard to blood pressure measurements, the follow-up rate was 92% in the GBP group and 92% in the VBG/B group at 2 y, and 68% vs. 74%, respectively, at the 10 y follow-up. The follow-up rate in the control group was 81% and 62% at 2 y and 10 y, respectively. Baseline characteristics for each study cohort are given in Table 1 and 2.

### Examination procedures

Systolic blood pressure and Korotkoff phase 5 diastolic blood pressure (after 15 min in the supine position), body weight and height were measured at each study visit. Use of blood pressure lowering medications (antihypertensives) was assessed with a questionnaire. A user of antihypertensives was defined as someone who, on a daily basis, took one or more medications included under the following codes: C02 (antihypertensives), C03 (diuretics), C07 (beta blocking agents), C08 (calcium channel blockers) or C09 (agents acting on the renin-angiotensin system) in the Anatomic Therapeutic Chemical (ATC) classification system. A user of diuretics was defined as someone who, on a daily basis, took a medication included under the ATC code: CO3 (diuretics). The subjects were instructed to collect urine over 24 h prior to the baseline examination, the 2 y follow-up visit and the 10 y follow-up visit, and to bring it to the examination site the next day. With regard to urine collections, the follow-up rate was 90% in the GBP group and 91% in the VBG/B group at 2 y, and 66% vs. 72%, respectively, at the 10 y follow-up. The follow-up rate in the control group was 81% and 61% at 2 y and 10 y, respectively. The volume of the urine was noted and a 200-mL sample was transferred to the Central Laboratory of Sahlgrenska University Hospital (accredited according to European Norm EN45001). Urinary and serum Na^+^ and K^+^ were measured by an ion-selective electrode (Modular P, Roche Diagnostics Scandinavia AB, Bromma, Sweden). Urinary and serum creatinine was measured by enzymatic photometry (Modular P, Roche Diagnostics Scandinavia AB). Twenty-four hour urinary excretions of sodium (U-Na^+^), potassium and creatinine were calculated from these data and the recorded 24 h urine output (U-Volume). The schedule, questionnaire, blood pressure and anthropometric measurements, and laboratory examinations were identical for GBP, VBG/B and control subjects.

### Statistical analysis

The Students *t* test compared continuous variables at baseline, and the chi-square test was used for all comparisons of proportions. All changes over time were calculated as the difference between individual values at baseline and values at the 2 y and 10 y follow-up visits. Adjustments for baseline differences between the cohorts (Table 1 & 2) were made using multiple linear regression, taking into account sex, age and baseline BMI. Only adjusted blood pressure changes are reported. In some analyses (as indicated in text and legends), BMI change was added as a covariate to the multiple regression model in order to compare subjects with equal BMI changes after GBP and VBG/B surgery. Changes in 24 h urinary excretion of creatinine were included as a covariate in order to adjust for potential between-group differences in the completeness of 24 h urine collections. All *P*-values are two tailed. Means and 95% confidence intervals (CI) are given in the text and figures, unless otherwise stated. Statistical significance was set at *P*<0.05. The statistical analyses were carried out using SPSS, version 18 (SPSS Inc., Chicago, Illinois).

## Results

### Arterial pressure was markedly reduced over the long term by gastric bypass

**Figure 1:**
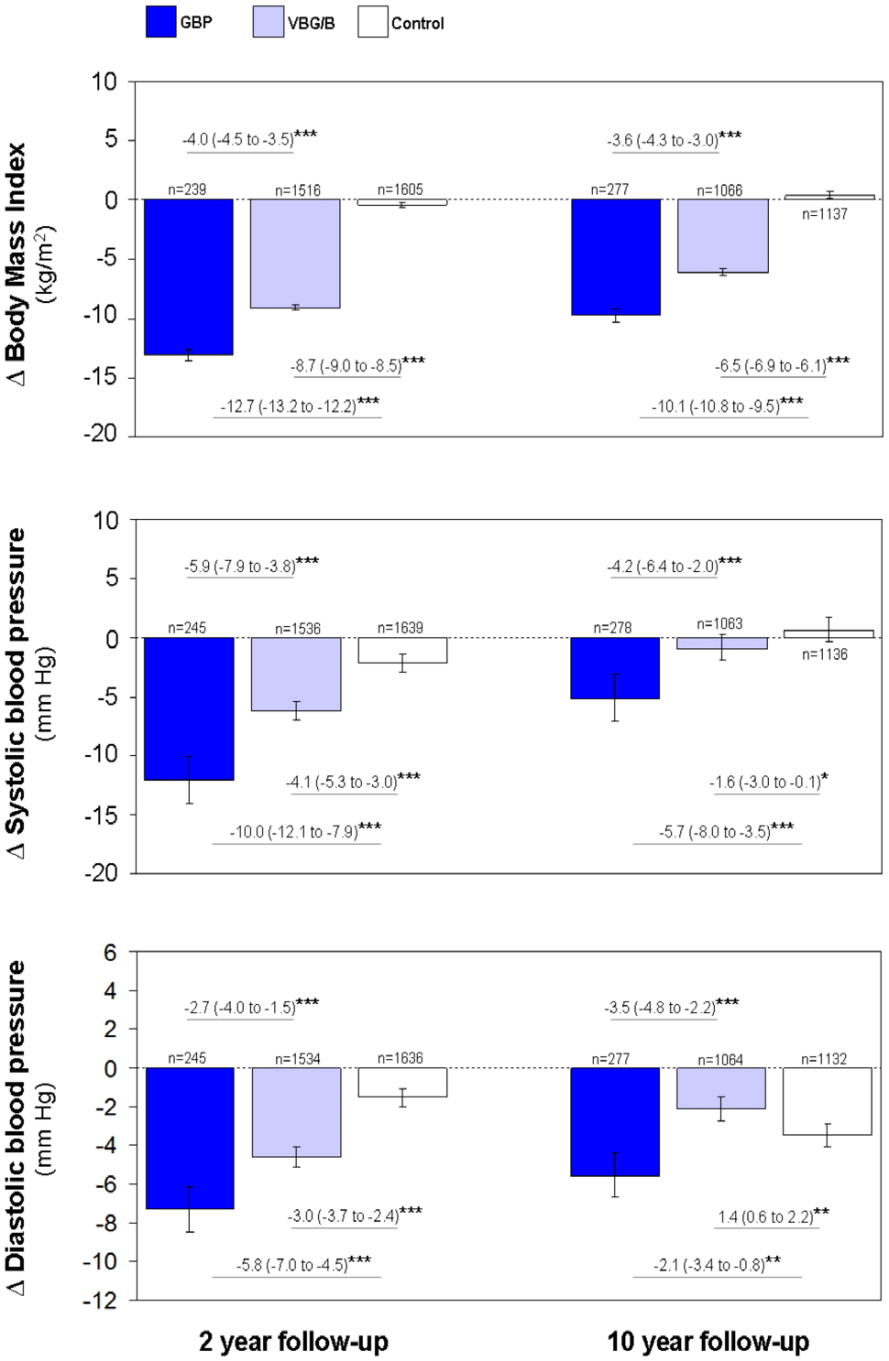
Changes in BMI and blood pressure following different types of bariatric surgery. Changes in body mass index (BMI) and blood pressure after gastric bypass surgery (GBP), after pure restrictive bariatric surgery (VBG/B) and in non-operated obese controls at the 2 y and 10 y follow-up visits. Data are mean values adjusted for sex, age, baseline BMI and the baseline level of the respective variables. The bars represent the 95% confidence intervals. Differences between groups are given as mean (95% confidence intervals). *P<0.05, **P<0.01 and ***P<0.001.

Weight reduction following GBP was greater than after VBG/B, while non-surgical treatment was not associated with any significant effect (Figure 1, upper panel). The systolic pressure in the GBP cohort had decreased by −12.1 (−14.0 to −10.1) and −5.1 (−7.1 to −3.1) mm Hg, and the diastolic pressure by −7.3 (−8.5 to −6.2) and −5.6 (−6.7 to −4.4) mm Hg, compared to baseline, at the 2 y and 10 y follow-ups, respectively (Figure 1, middle and lower panels). The VBG/B cohort also demonstrated reduced arterial pressure at the 2 y follow-up, but this decrease was significantly smaller than that in the GBP-treated patients. Systolic blood pressure in the VBG/B cohort at the 10 y follow-up had decreased slightly compared to controls, whereas diastolic pressure had actually decreased more in the non-operated controls. The weight reduction was linearly associated with changes in blood pressure after VBG/B and in the controls. Interestingly, such an association was less evident after 2 y, and absent at the 10 y follow up visit in the GBP cohort (Supporting information Figure S2).

After 2 y, the proportions of patients taking antihypertensives did not differ between the GBP (27%) and VBG/B (31%) cohorts. However, both these proportions were significantly lower (*P*<0.001) than in the control cohort (43%). At the 10 y visit, the proportion of patients using antihypertensives was significantly lower in the GBP group (35%) as compared to both the VBG/B (45%, *P*<0.01) and the controls (53%, *P*<0.001).

### Gastric bypass exerted a weight-independent diuretic effect

**Figure 2:**
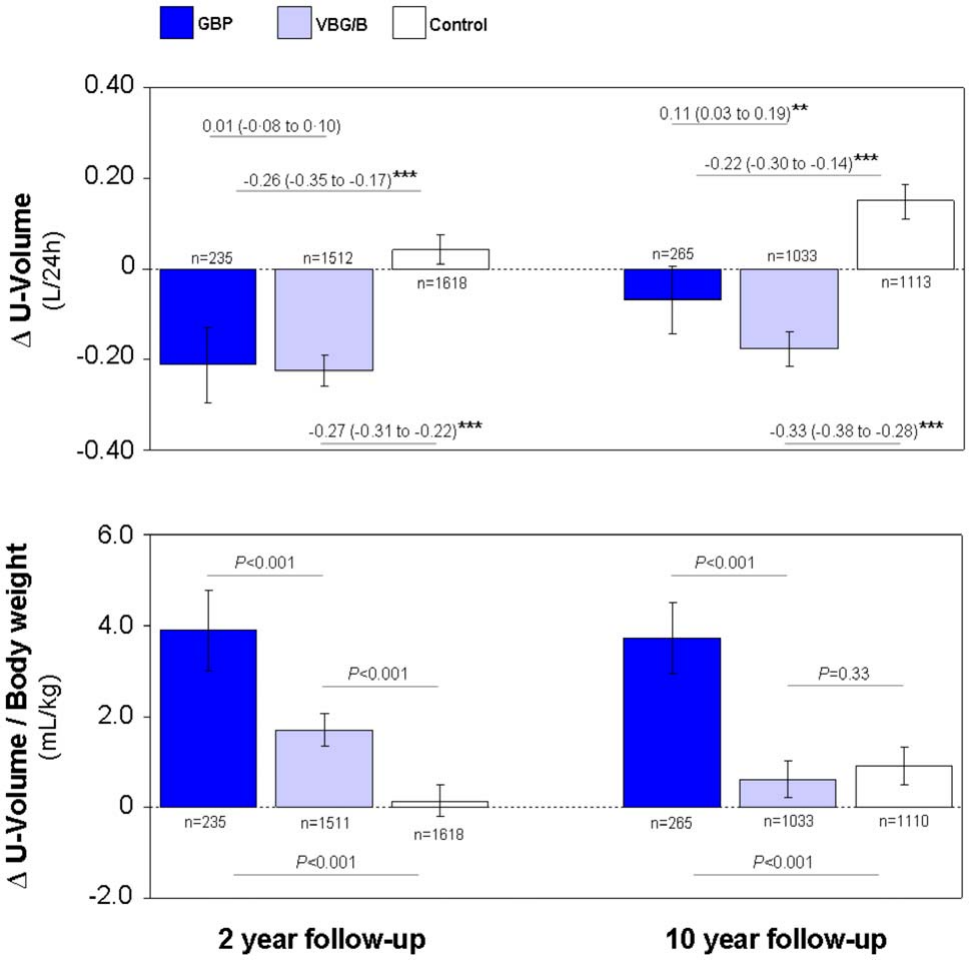
Diurnal urinary output after different types of bariatric surgery. Diurnal urinary output (U-Volume) in absolute values (upper panel) and in relation to body weight (lower panel) after gastric bypass surgery (GBP), after pure restrictive bariatric surgery (VBG/B) and in non-operated obese controls. Changes from baseline (Δ) at the 2 y and 10 y follow-up visits are displayed. Data are mean values adjusted for sex, age, baseline BMI and the baseline level of the respective variables. The bars represent the 95% confidence intervals. Differences between groups (upper panel) are given as mean (95% confidence intervals).*P<0.01 and ***P<0.001.

Diurnal urinary outputs (U-Volume) in absolute values were reduced in both the GBP and the VBG/B cohorts (Figure 2, upper panel). When related to body weight, GBP patients exhibited higher urinary volumes at both the 2 y and 10 y follow-up visits (Figure 2, lower panel). A similar effect, although of less than half the magnitude in the GBP group, was present in the VBG/B cohort after 2 y, but not at the 10 y follow-up.

The diuretic effect of GBP was further quantified by direct comparison of the two bariatric techniques after adjustment for BMI reduction. The 24 h urinary output was +0.10 and +0.17 L larger after GBP as compared to VBG/B at the 2 y and 10 y follow-ups, respectively (Table 3 and 4). The proportion of patients using diuretics at the 2 y and 10 y follow-ups did not differ between the GBP (10% and 17%, respectively) and the VBG/B cohorts (13% and 19%, respectively). Also after exclusion of patients using diuretics the 24 h urinary output was +0.10 (95% CI 0.01–0.20) and +0.17 (0.08 to 0.26) L larger after GBP as compared to VBG/B at the 2 y and 10 y follow-ups, respectively.

**Table 3:** Weight reduction independent changes from baseline after GBP and VBG/B at the 2 year follow up (multiple linear regression analyses).

	aUnadjusted change		bWeight reduction adjusted change		cDifference between groups in b
	GBP(n = 235)	VBG/B (n = 1512)	GBP	VBG/B	
**Urinary (24 h)**					
Volume (L)	0.23 (0.73)	−0.20 (0.81)	−0.11	−0.21	0.10 (0.004 to 0.19)*
Sodium (mmol)	−55 (105)	−53 (114)	−25	−45	19 (7 to 32)**
Daily salt intake(g)#	−3.2 (6.1)	−3.1 (6.7)	−1.5	−2.6	1.1 (0.4 to 1.9)**
Potassium (mmol)	−21 (31)	−17 (38)	−17	−14	−3 (−7 to 1)
Sodium:Potassium	0.1 (1.6)	−0.1 (1.4)	00.04	−0.1	0.5 (0.3 to 0.7)***
Creatinine (mmol)	−2.8 (3.7)	−2.4 (7.1)	−2.0	−1.7	−0.3 (−1.2 to 0.6)
**Serum**					
Sodium (mM)	0.8 (2.9)	1.1 (3.2)	01.04	01.00	0.4 (0.1 to 0.7)*
Potassium (mM)	−0.08 (0.33)	−0.08 (0.35)	−0.04	−0.07	0.03 (0.01 to 0.07)
Creatinine (µM)	−1.0 (8.5)	−0.3 (9.1)	−0.3	00.01	0.4 (−0.7 to 1.5)

a **:** Unadjusted difference between year 2 and baseline within each group. Minus signs denote reductions. Means (±SD) ^b^Difference between year 2 and baseline within each group after adjustment for change in body mass index (BMI), sex, age, baseline BMI and the baseline level of the respective variables. Minus signs denote reductions. Mean values.

b **:** Difference between year 2 and baseline after adjustment for change in body mass index (BMI), sex, age, baseline BMI and the baseline level of the respective variables. Minus signs denote reductions. Mean values.

c : Difference between gastric bypass surgery (GBP) and pure restrictive bariatric surgery (VBG/B) after adjustment for BMI change, sex, age, baseline BMI and the baseline level of the respective variables. Minus signs denote larger reductions in GBP compared to VBG/B group. Figures between brackets denote 95% confidence interval.

# Daily salt intake was calculated by multiplying urinary sodium values by 0.0585 (molecular weight of NaCl: 58.5).

* P<0.05,

** P<0.01 and ***P<0.001 for test of difference between the GBP group and the VBG/B group in adjusted changes, using multiple linear regression.

**Table 4:** Weight reduction independent changes from baseline after GBP and VBG/B at the 10 year follow up (multiple linear regression analyses).

	aUnadjusted change		bWeight reduction adjusted change		cDifference between groups in b
	GBP (n = 265)	VBG/B (n = 1033)	GBP	VBG/B	
**Urinary (24 h)**					
Volume (L)	−0.11 (0.79)	−0.17 (0.75)	0.00	−0.17	0.17 (0.09 to 0.26)***
Sodium (mmol)	−49 (109)	−52 (104)	−25	−45	20 (10 to 30)***
Daily salt intake(g)#	−2.9 (6.4)	−3.0 (6.1)	−1.5	−2.7	1.2 (0.6 to 1.8)***
Potassium (mmol)	−16 (31)	−19 (34)	−12	−17	5 (2 to 9)**
Sodium:Potassium	−0.06 (1.6)	0.02 (1.4)	0.09	0.03	0.06 (−0.11 to 0.24)
Creatinine (mmol)	−3.0 (4.3)	−2.9 (3.9)	−3.1	−2.6	0.4 (−0.1 to 1.0)
**Serum**					
Sodium (mM)	1.9 (3.0)	1.6 (2.7)	2.1	1.4	0.7 (0.4 to 1.0)***
Potassium (mM)	−0.02 (0.34)	−0.05 (0.35)	0.06	−0.04	0.09 (0.05 to 0.14)***
Creatinine (µM)	−12.9 (16.3)	−2.1 (26.9)	−10.8	−0.5	−10.3 (−13.7 to −6.9)***

a **:** Unadjusted difference between year 10 and baseline within each group. Minus signs denote reductions. Means (±SD) ^b^Difference between year 10 and baseline within each group after adjustment for change in body mass index (BMI), sex, age, baseline BMI and the baseline level of the respective variables. Minus signs denote reductions. Mean values.

b **:** Difference between year 10 and baseline after adjustment for change in body mass index (BMI), sex, age, baseline BMI and the baseline level of the respective variables. Minus signs denote reductions. Mean values.

c : Difference between gastric bypass surgery (GBP) and pure restrictive bariatric surgery (VBG/B) after adjustment for BMI change, sex, age, baseline BMI and the baseline level of the respective variables. Minus signs denote larger reductions in GBP compared to VBG/B group. Figures between brackets denote 95% confidence interval.

# Daily salt intake was calculated by multiplying urinary sodium values by 0.0585 (molecular weight of NaCl: 58.5).

* P<0.05,

** P<0.01 and ***P<0.001 for test of difference between the GBP group and the VBG/B group in adjusted changes, using multiple linear regression.

### Urinary output was associated with BP changes only after gastric bypass

Changes in systolic and diastolic blood pressures (BP) were negatively associated with the change in diurnal urinary output (*P*<0.01 at the 2 y follow-up and *P*<0.05 at the 10 y follow-up, respectively) after gastric bypass (Supporting information Figure S3). No associations between blood pressure and delta urinary volume were observed in the VBG/B or control groups.

### Salt excretion was higher after gastric bypass than after restrictive surgery

**Figure 3:**
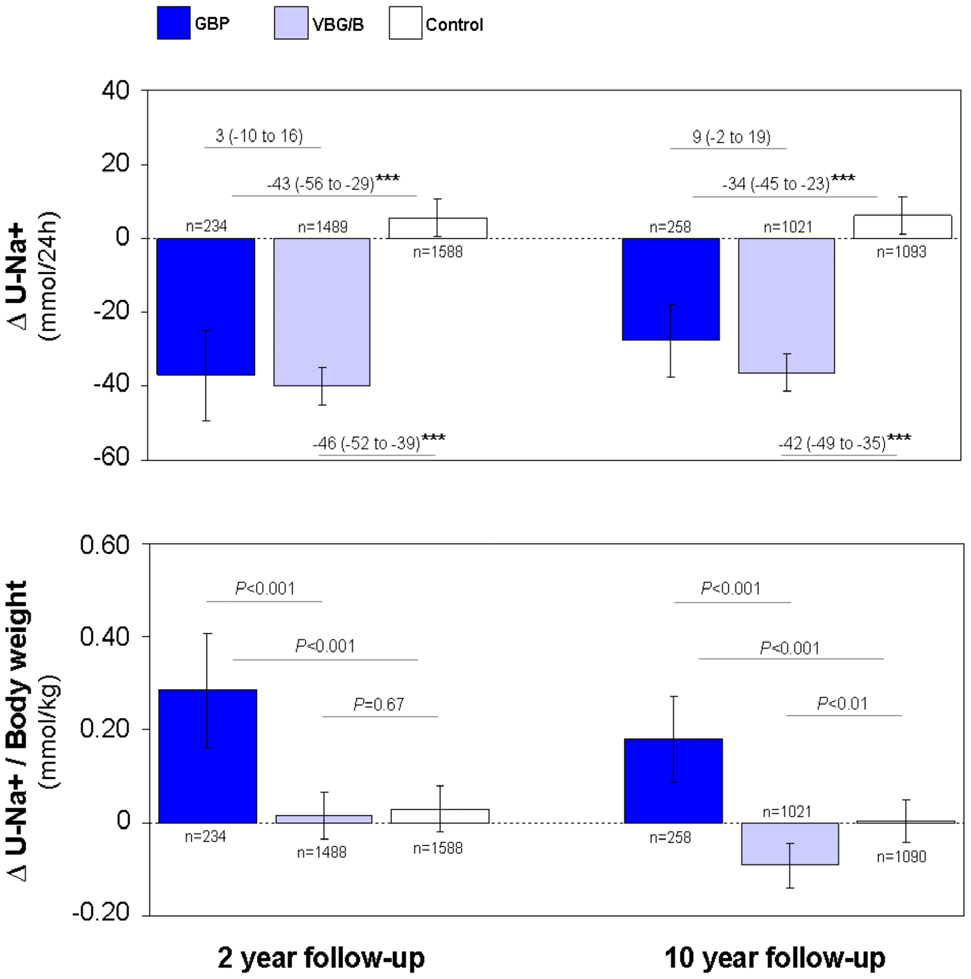
Diurnal urinary excretion of sodium after different types of bariatric surgery. Diurnal urinary excretion of sodium (U-Na^+^) in absolute values (upper panel) and in relation to body weight (lower panel) after gastric bypass surgery (GBP), after pure restrictive bariatric surgery (VBG/B) and in non-operated obese controls. Changes from baseline (Δ) at the 2 y and 10 y follow-up visits are displayed. Data are mean values adjusted for sex, age, baseline BMI and the baseline level of the respective variables. The bars represent the 95% confidence intervals. Differences between groups (upper panel) are given as mean (95% confidence intervals). ***P<0.001.

Diurnal urinary excretion of sodium (U-Na^+^) at the 2 y and 10 y follow-ups had decreased in the GBP and VBG/B cohorts and had increased slightly in the controls (Figure 3, upper panel). When related to body weight, U-Na^+^ had increased in the GBP group but was unchanged in the VBG/B group and in the non-operated controls at 2 y (Figure 3, lower panel). At the 10 y follow-up, U-Na^+^ in relation to body weight remained increased in the GBP cohort but had decreased in the VBG/B group and was unchanged in obese controls.

A BMI reduction-adjusted quantitative analysis showed that the 24 h urinary excretion of sodium was +19 and +20 mmol higher in the GBP group than in the VBG/B group at the 2 y and 10 y follow-ups, respectively (Table 2). Because urinary Na^+^ output over 24 h equals Na^+^ intake, daily salt (NaCl) intake could be calculated: GBP subjects were calculated to consume 1.1 g (0.4 to 1.9) and 1.2 g (0.6 to 1.8) more salt on a daily basis than did the VBG/B subjects with equal BMI changes 2 y and 10 y after surgery, respectively.

Systolic and diastolic blood pressures were positively associated with salt intake in the VBG/B and control groups at 2 y and 10 y, respectively (data not shown). However, no associations between blood pressure changes and salt excretion were observed after GBP.

## Discussion

Bariatric surgery was originally developed for weight reducing purposes. Today we know that these gastrointestinal interferences also markedly reduces the risk for cardiovascular disease as shown by an estimated 40% reduction in 10 y Framingham risk score [5]. Furthermore, Heneghan et al showed that bariatric surgery is associated with a 68% resolution/reduction of obesity associated hypertension [5]. However, the mean follow up time of this analysis was only 34 months and studies over longer periods (*ie.* 8 to 10 y) are few and usually uncontrolled [5], [6]. The present investigation, being an *ad hoc-*analysis based on the controlled prospective SOS study [4], [15], provides the first longterm direct comparison between conventionally treated (*ie.* dietary advice, life style changes, pharmacology etc) obese patients and those treated surgically with either GBP or VBG/B. Previously published meta-analyses have shown that a pharmacological reduction of diastolic pressure by 5 mm Hg considerably reduces the risks of stroke and ischemic heart disease [7]. Interestingly, a blood pressure reduction of this order of magnitude was in the present study recorded only in the GBP cohort at the 10 y follow-up and despite a relatively low usage of antihypertensives. It is well known that weight reduction *per se* is associated with a decrease in blood pressure mainly due to the improvement of renal sodium retention that is commonly associated to obesity [16], [17]. Increased exercise and a diet with low sodium and calorie contents can reduce blood pressure in a weight dependent fashion [13], [18]. However, the long-term compliance with these types of life style changes is low and there is eventually a relapse to overweight and increased blood pressure. Bariatric surgery is currently the only evidence-based treatment for maintaining a reduction in body weight [3].

Indeed, at the 2 y follow-up in the present study, the two bariatric surgery cohorts exhibited reduced BMI and a reduced arterial blood pressure compared to the non-operated controls. However, at the 10 y follow-up in VBG/B patients, blood pressure had returned near to preoperative levels despite a reduction in body weight. One explanation may be that an increased inclination for social interaction following weight reduction creates iterated situations with a psychosocial stimulation of the sympathetic nervous system, in turn increasing blood pressure [17]. Another possibility is that restrictive bariatric surgery induces a dietary pattern promoting hypertension [19].

In contrast to restrictive bariatric surgery, GBP was followed by a marked decrease in blood pressure also at the 10 y follow-up. This finding suggests that GBP interferes with blood pressure control via at least 2 principles: an initial transient blood pressure reduction related to weight loss *per se* (similar as after VBG/B), and a more long term mechanism unrelated to weight loss. This interpretation is supported by the present observation that arterial blood pressure was markedly more depressed after GBP than after VBG/B, already after a period of only 2 y. Actually, a weight-independent blood pressure reducing effect of GBP has previously been reported as early as week one postoperatively, thus before any significant weight loss [20]. Based on these observations it is plausible to assume that the exclusion of the gastroduodenum, or the direct loading of undigested food into the jejunum, added or removed a blood pressure regulating factor acting in parallel with the depressor effect of weight loss (Supporting information Figure S2).

In analysing diurnal urinary volumes, we found that the GBP patients excreted more urine per day than weight-matched patients treated with restrictive bariatric surgery. Furthermore, regression analysis demonstrated that changes in urinary output were linearly associated with blood pressure changes only in the GBP cohort (Supporting information Figure S3), indicating that blood pressure reduction following gastric bypass can be attributed to a diuretic action. It can perhaps be questioned whether a chronic enhancement of diuresis by only 100 to 200 mL per day explains the lowered blood pressure in the GBP cohort. Interestingly, the users of diuretics compared to non-users in the non-operated cohort had a similar difference in urinary output as the one observed between GBP and VBG/B (Supporting information Table S1). These results indicate that the GBP-associated diuretic action is of clinical relevance with regard to blood pressure control.

The mechanisms behind the diuretic action following GBP are obscure. Bueter *et al.* recently demonstrated that an oral salt load in rats operated with GBP was followed by an increased urine output and natriuresis as well as increased water intake. However, these authors could not distinguish between if the increased diuresis was a primary mechanism, or if it was secondary to blood volume expansion following drinking [21]. In the present study, 24 h natriuresis was higher after GBP, whereas potassium excretion was more or less constant, suggesting a primary natriuretic effect. The finding that the serum sodium concentration was elevated in the GBP group speaks, however, against such an action (Table 2) [22].

We found that the calculated salt intake was more than +1 g higher per day after GBP compared to salt intake in the patients treated with restrictive bariatric surgery. Intuitively, high salt intake should increase blood pressure [12]–[14]. However, this was not the case in the present study indicating that GBP patients lower their blood pressure despite an increased consumption of salty foods. It may be speculated that GBP increases both salt appetite and natriuresis explaining the slightly increased serum sodium concentrations observed in the present study. By use of surveys, Tichansky *et al*. showed that GBP patients were more likely to develop an increased taste/affinity for salty foods than gastric banding patients who where more likely to develop an increased taste/affinity for sweet foods [23]. Hence, our findings support the existence of a sodium sensor in the human upper gut [24]–[26], that normally inhibits salt appetite and influence natriuresis, being “bypassed” following GBP. The involved gastrointestinal signalling mechanisms remains to be clearified.

The present study has some obvious limitations: The cohort analysis was performed *ad hoc* and interpretations must be made with caution because the SOS study was originally designed to compare bariatric surgery *per se* with conventionally treated obese patients and the study power was determined using mortality as the primary outcome variable [15]. Another potential source of error in the present study is the 24 h sampling of urine. As the urine was collected by the study participants themselves, sampling errors related to over- or under-collections must be considered. However, there is little reason to believe that any of the surgery groups would be more likely to provide over- or under-collections. In addition, the differences in diurnal urine output and sodium excretion between GBP- and VBG/B subjects remained significant after adjusting these analyses for 24 h urinary excretion of creatinine (data not shown). Another potential limitation of the study is that the cohort representing ‘restrictive’ bariatric surgery consists of two types of surgical procedures: VBG (n = 1189 and n = 843 at the 2 y and 10 y visit, respectively) and gastric banding (n = 328 and n = 202). These two operations have some obvious technical differences that might have influenced the primary outcome variables (BMI, arterial pressure, use of antihypertensives/diuretics, diurnal urinary volume, estimated salt intake). However, when a subgroup comparison was performed only one significant difference appeared: the diastolic pressure had decreased more after gastric banding at the 2 y visit (Supporting information Table S2). As this difference (≈1,2 mmHg) is of minor clinical relevance the merger of the two operations into a single cohort seems to be justified.

In conclusion: Gastric bypass is associated with a longstanding reduction in both systolic and diastolic blood pressures and an increased diuresis not related to weight loss. Despite a greater blood pressure reduction, the daily salt consumption is higher after GBP than after restrictive bariatric surgery.

## Supporting Information

Figure S1 **Diagram showing the three study cohorts as organised from the Swedish Obese Study.**
(PDF)

Figure S2 **Linear relationship between blood pressure changes and changes in BMI after gastric bypass surgery (GBP), after pure restrictive bariatric surgery (VBG/B) and in non‐operated obese controls at the 2 y and 10 y follow‐up visits.** Regression lines and beta values (unadjusted) illustrate results of simple linear regression analysis, while adjusted beta values and P‐values are results of the multiple linear regression analysis adjusted for change in daily salt intake, as well as for sex, age and baseline BMI.
(PDF)

Figure S3 **Linear relationship between blood pressure changes and changes in diurnal urinary output (U**-**Volume) after gastric bypass surgery (GBP), after pure restrictive bariatric surgery (VBG/B) and in non‐operated obese controls at the 2 y and 10 y follow**-**up visits.** Regression lines and beta values (unadjusted) illustrate results of simple linear regression analysis, while adjusted beta values and P‐values are results of multiple linear regression analysis adjusted for both BMI change and change in daily salt intake, as well as for sex, age and baseline BMI.
(PDF)

Table S1 **Urinary and serum variables in users and non-users of diuretics among obese controls at the baseline examination.**
(PDF)

Table S2 **Changes from baseline after vertical banded gastroplasty (VBG) and gastric banding at 2 and 10 years (multiple linear regression analyses).**
(PDF)
