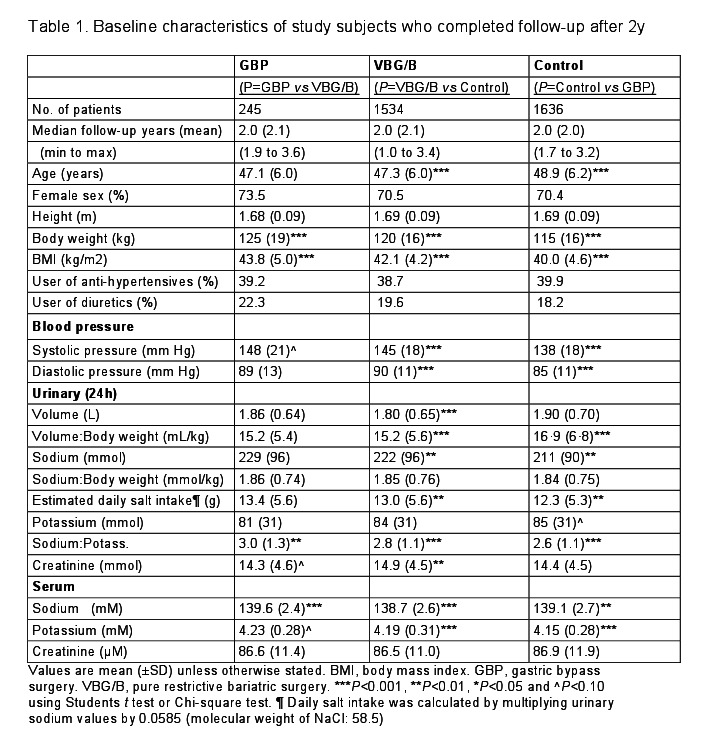# Correction: Gastric Bypass Surgery Is Followed by Lowered Blood Pressure and Increased Diuresis - Long Term Results from the Swedish Obese Subjects (SOS) Study

**DOI:** 10.1371/annotation/88dbb5b7-3f7e-4b26-a5b6-f020d33f0182

**Published:** 2013-05-22

**Authors:** Peter Hallersund, Lars Sjöström, Torsten Olbers, Hans Lönroth, Peter Jacobson, Ville Wallenius, Ingmar Näslund, Lena M. Carlsson, Lars Fändriks

There were errors in Table 1. The correct version of Table 1 is available here: 

**Figure:**