# Supplementary material for: Gastric Bypass Surgery Is Followed by Lowered Blood Pressure and Increased Diuresis - Long Term Results from the Swedish Obese Subjects (SOS) Study
Source: PLoS One. 2012 Nov 29;7(11):e49696. doi: 10.1371/journal.pone.0049696 (PMC3510228; doi:10.1371/journal.pone.0049696)
Supplement: Figure S1 — Diagram showing the three study cohorts as organised from the Swedish Obese Study. (PDF) [file pone.0049696.s001.pdf]

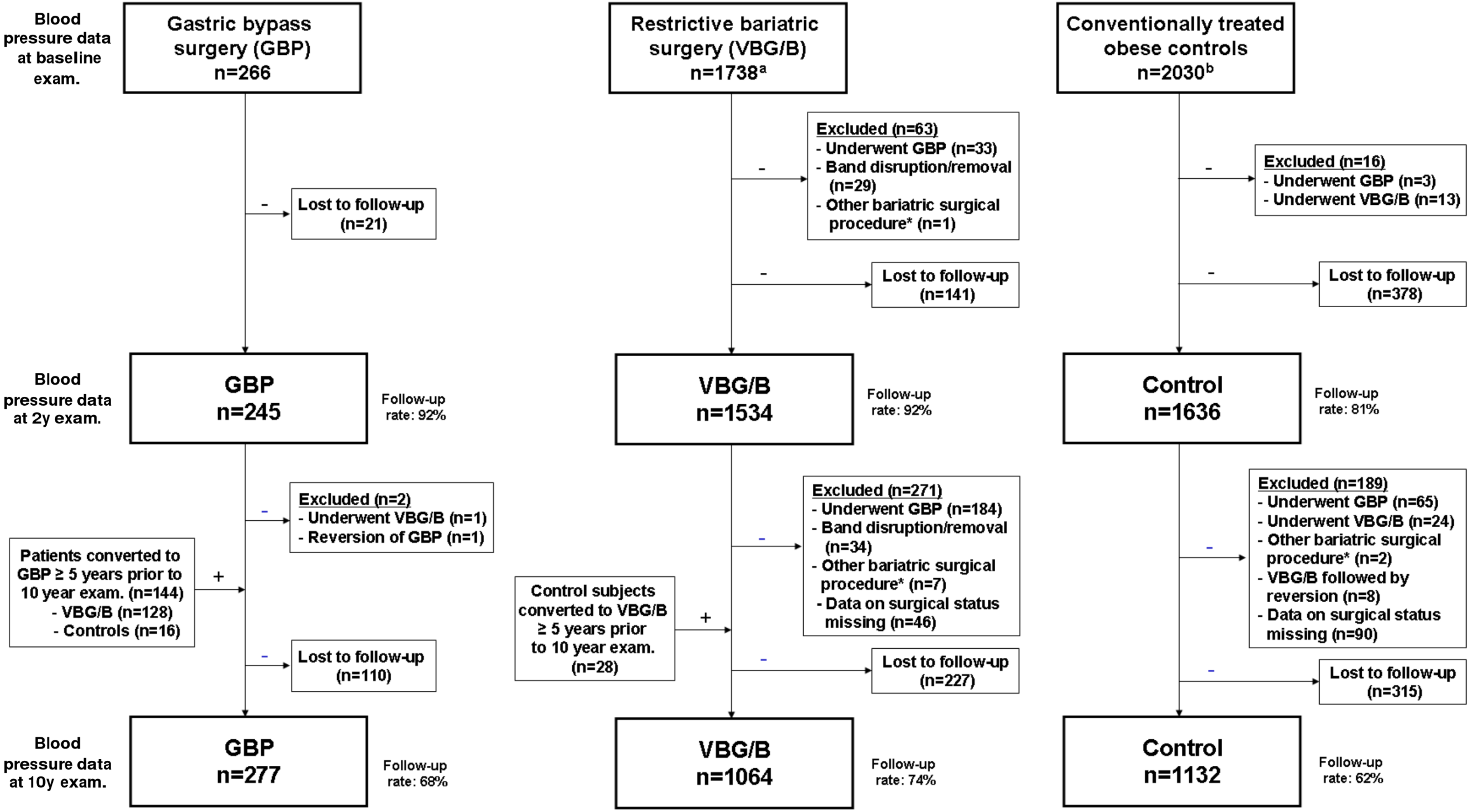


[a]Data on diastolic blood pressure were missing for 6 patients (0.3% of VBG/B subjects) in the Swedish obese subjects (SOS) study.
[b]Data on diastolic blood pressure were missing for 7 patients (0.3% of control patients) in the SOS study.
*Other bariatric surgical procedures included gastric sleeve, duodenal switch and jejuno-intestinal bypass.
