# Supplementary figures and images for: Gastric Bypass Surgery Is Followed by Lowered Blood Pressure and Increased Diuresis - Long Term Results from the Swedish Obese Subjects (SOS) Study
Source: PLoS One. 2012 Nov 29;7(11):e49696. doi: 10.1371/journal.pone.0049696 (PMC3510228; doi:10.1371/journal.pone.0049696)

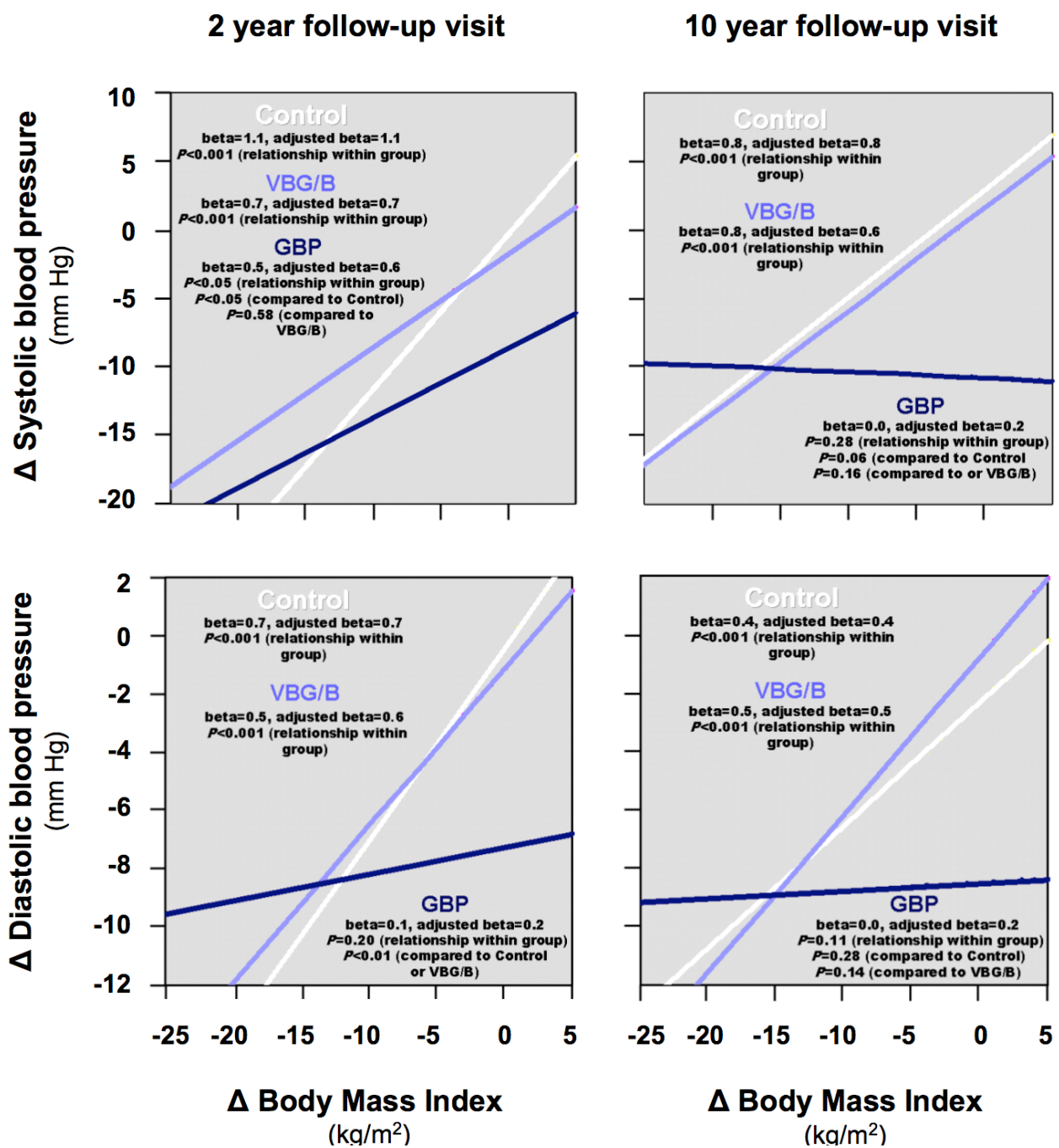

Figure S2

Supplement: Figure S2 — Linear relationship between blood pressure changes and changes in BMI after gastric bypass surgery (GBP), after pure restrictive bariatric surgery (VBG/B) and in non‐operated obese controls at the 2 y and 10 y follow‐up visits. Regression lines and beta values (unadjusted) illustrate results of simple linear regression analysis, while adjusted beta values and P‐values are results of the multiple linear regression analysis adjusted for change in daily salt intake, as well as for sex, age and baseline BMI. (PDF) [file pone.0049696.s002.pdf]

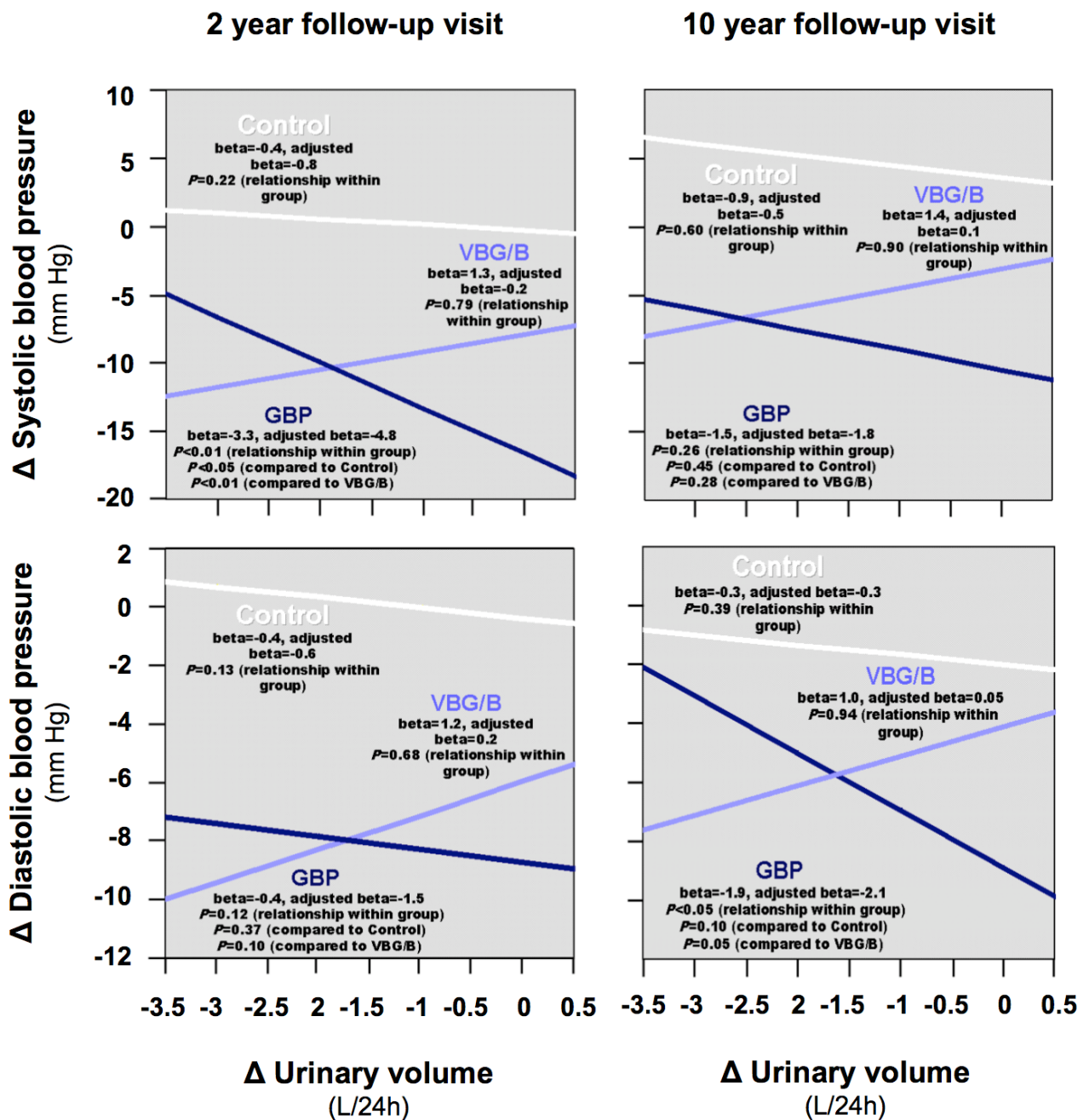

Figure S3

Supplement: Figure S3 — Linear relationship between blood pressure changes and changes in diurnal urinary output (U-Volume) after gastric bypass surgery (GBP), after pure restrictive bariatric surgery (VBG/B) and in non‐operated obese controls at the 2 y and 10 y follow-up visits. Regression lines and beta values (unadjusted) illustrate results of simple linear regression analysis, while adjusted beta values and P‐values are results of multiple linear regression analysis adjusted for both BMI change and change in daily salt intake, as well as for sex, age and baseline BMI. (PDF) [file pone.0049696.s003.pdf]
