# Supplementary material for: Gastric Bypass Surgery Is Followed by Lowered Blood Pressure and Increased Diuresis - Long Term Results from the Swedish Obese Subjects (SOS) Study
Source: PLoS One. 2012 Nov 29;7(11):e49696. doi: 10.1371/journal.pone.0049696 (PMC3510228; doi:10.1371/journal.pone.0049696)
Supplement: Table S1 — Urinary and serum variables in users and non-users of diuretics among obese controls at the baseline examination. (PDF) [file pone.0049696.s004.pdf]

Supporting information table S1:

Urinary and serum variables in users and non-users of diuretics among obese controls at the baseline examination

	Users of Diuretics (UD) (n=239)	All control subjects not using diuretics (n=1237)	Only controls using other types of BP medications (n=285)	Difference comparing UD to all controls not using diuretics	Difference comparing UD to controls using other types of BP medications
	mean (±SD)	mean (±SD)	mean (±SD)	mean (95% CI)	mean (95% CI)
**Urinary (24h)**					
Volume (L)	2.07 (0.68)	1.90 (0.76)	1.92 (0.73)	0.18 (0.07 to 0.28)***	0.15 (0.03 to 0.27)*
Sodium (mmol)	224 (96)	204 (86)	221 (92)	20 (6 to 33)**	3 (-13 to 19)
Estimated daily salt intake[#] (g)	13.1 (5.6)	11.9 (5.0)	12.9 (5.4)	1.1 (0·4 to 1.9)**	0.2 (-0.8 to 1.1)
Potassium (mmol)	84 (30)	83 (31)	87 (32)	1 (-4 to 5)	-3 (-8 to 2)
Sodium:Potassium	2.8 (1.1)	2.6 (1.0)	2.7 (1.1)	0.2 (0.1 to 0.4)**	0.1 (-0.1 to 0.3)
Creatinine (mmol)	13.6 (4.2)	13.9 (4.2)	14.4 (4.3)	-0.4 (-0.9 to 0.2)	-0.9 (-1.6 to -0.1)*
**Serum**					
Sodium (mmol/L)	139.4 (2.8)	139.1 (2.7)	138.8 (2.6)	0.3 (-0.05 to 0.7)$^{P=0.09}$	0.6 (0.1 to 1.1)*
Potassium (mmol/L)	4.04 (0.34)	4.18 (0.28)	4.16 (0.27)	-0.14 (-0.19 to -0.09)***	-0.12 (-0.18 to -0.07)***
Creatinine (μmol/L)	89.6 (15.3)	86.6 (11.3)	89.0 (12.4)	3.0 (1.0 to 5.1)**	0.6 (-1.8 to 2.9)
**Blood pressure**					
Systolic pressure (mm Hg)	144 (18)	137 (18)	146 (20)	6.9 (4.4 to 9.5)***	-1.3 (-4.6 to 1.9)
Diastolic pressure (mm Hg)	87 (11)	85 (11)	89 (11)	2.5 (1.0 to 4.0)***	-1.4 (-3.2 to 0.4)

A user of diuretics was defined as someone who, on a daily basis, took a medication included under the code: CO3 (diuretics) in the Anatomic Therapeutic Chemical (ATC) classification system.

Estimated daily salt intake was calculated by multiplying urinary sodium values by 0.0585 (molecular weight of NaCl: 58.5). CI denotes confidence interval.

**P*<0.05 ***P*<0.01 and ****P*<0.001 comparing users and non-users of diuretics with Students *t* test
