# Supplementary material for: Gastric Bypass Surgery Is Followed by Lowered Blood Pressure and Increased Diuresis - Long Term Results from the Swedish Obese Subjects (SOS) Study
Source: PLoS One. 2012 Nov 29;7(11):e49696. doi: 10.1371/journal.pone.0049696 (PMC3510228; doi:10.1371/journal.pone.0049696)
Supplement: Table S2 — Changes from baseline after vertical banded gastroplasty (VBG) and gastric banding at 2 and 10 years (multiple linear regression analyses). (PDF) [file pone.0049696.s005.pdf]

Supporting information table S2:

Changes from baseline after vertical banded gastroplasty (VBG) and gastric banding at 2 and 10 years (multiple linear regression analyses).

	Changes (Δ) at 2 year follow-up visit			Changes (Δ) at 10 year follow-up visit		
	Adjusted change[a] mean		P	Adjusted change[a] mean		P
	VBG (n=1189)	Gastric banding (n=328)		VBG (n=843)	Gastric banding (n=202)	
BMI (kg/m^2)	-9.5	-9.1	0.14	-6.5	-6.1	0.83
Systolic pressure (mm Hg)	-7.4	-9.3	0.055	-3.3	-2.6	0.54
Diastolic pressure (mm Hg)	-5.7	-6.9	0.04	-3.8	-3.8	0.37
User of anti-hypertensives (%)	31	31	0.99	45	40	0.17
User of diuretics (%)	13	14	0.53	19	19	0.95
Diurnal urinary volume (L)	-0.20	-0.22	0.51	-0.15	-0.12	0.46
Estimated daily salt intake[#] (g)	-2.6	-2.2	0.27	-2.5	-2.3	0.54

[a] Difference between year 2 or 10 and baseline within each group after adjustment for change in body mass index (BMI), sex, age, baseline BMI and the baseline level of the respective variables. Urinary volume and salt intake are also adjusted for BMI change. Minus signs denote reductions.

[#] Estimated daily salt intake was calculated by multiplying urinary sodium values by 0.0585 (molecular weight of NaCl: 58.5).
